# *In Vivo* Human Left-to-Right Ventricular Differences in Rate Adaptation Transiently Increase Pro-Arrhythmic Risk following Rate Acceleration

**DOI:** 10.1371/journal.pone.0052234

**Published:** 2012-12-20

**Authors:** Alfonso Bueno-Orovio, Ben M. Hanson, Jaswinder S. Gill, Peter Taggart, Blanca Rodriguez

**Affiliations:** 1 Department of Computer Science, Computational Biology Group, University of Oxford, Oxford, United Kingdom; 2 Department of Mechanical Engineering, University College London, London, United Kingdom; 3 Guy’s and St. Thomas’ Hospital, London, United Kingdom; 4 The Neurocardiology Research Unit, University College Hospital, London, United Kingdom; University of Otago, New Zealand

## Abstract

Left-to-right ventricular (LV/RV) differences in repolarization have been implicated in lethal arrhythmias in animal models. Our goal is to quantify LV/RV differences in action potential duration (APD) and APD rate adaptation and their contribution to arrhythmogenic substrates in the *in vivo* human heart using combined *in vivo* and *in silico* studies. Electrograms were acquired from 10 LV and 10 RV endocardial sites in 15 patients with normal ventricles. APD and APD adaptation were measured during an increase in heart rate. Analysis of *in vivo* electrograms revealed longer APD in LV than RV (207.8±21.5 vs 196.7±20.1 ms; *P<0.05*), and slower APD adaptation in LV than RV (time constant τ^s^ = 47.0±14.3 vs 35.6±6.5 s; *P<0.05*). Following rate acceleration, LV/RV APD dispersion experienced an increase of up to 91% in 12 patients, showing a strong correlation (r^2^ = 0.90) with both initial dispersion and LV/RV difference in slow adaptation. Pro-arrhythmic implications of measured LV/RV functional differences were studied using *in silico* simulations. Results show that LV/RV APD and APD adaptation heterogeneities promote unidirectional block following rate acceleration, albeit being insufficient for establishment of reentry in normal hearts. However, in the presence of an ischemic region at the LV/RV junction, LV/RV heterogeneity in APD and APD rate adaptation promotes reentrant activity and its degeneration into fibrillatory activity. Our results suggest that LV/RV heterogeneities in APD adaptation cause a transient increase in APD dispersion in the human ventricles following rate acceleration, which promotes unidirectional block and wave-break at the LV/RV junction, and may potentiate the arrhythmogenic substrate, particularly in patients with ischemic heart disease.

## Introduction

Ventricular heterogeneity in repolarization is one of the most important contributors to the electrophysiological substrate leading to the occurrence of lethal arrhythmias such as ventricular fibrillation [1]–[5]. A large number of studies have demonstrated the complex spatio-temporal mechanisms that modulate ventricular heterogeneity in repolarization and pro-arrhythmic risk. Research using animal models has shown that both functional and structural differences between the left and the right ventricles (LV and RV) determine the spatio-temporal organization of ventricular fibrillation [6], [7], the generation of arrhythmias in sudden cardiac death syndromes [8], cardiac vulnerability to electric shocks [9], and epicardial repolarization gradient during global ischemia [10]. Even though LV/RV differences in action potential duration (APD) have been reported in several species, including canine [11], [12] and swine hearts [13], data in human are scarce. To the best of our knowledge, only one study by Ramanathan *et al.* [14] provides quantitative evidence of the interventricular differences in epicardial APD in 7 normal human subjects, using non-invasive imaging to reconstruct the repolarization pattern from body-surface signals.

Ventricular heterogeneity in repolarization and arrhythmic risk are known to increase with sudden changes in rate [15]–[17], due to the highly rate-dependent properties of the APD. *In vivo*, *in vitro* and *in silico* studies have shown that, following a rate increase, the human ventricular APD adapts in two distinct phases, starting with a fast decrease lasting only a few beats, then followed by a slow phase of the order of several minutes [18]–[20]. The dynamics of APD adaptation underlie the adaptation of the QT interval in the electrocardiogram [20]. Importantly, patients with protracted QT interval rate adaptation were associated with high arrhythmic risk [21], [22], supporting the importance of ventricular rate adaptation dynamics in arrhythmogenesis. However, very little is known about LV/RV differences in APD rate adaptation, which, if present, could contribute to increased interventricular dispersion in repolarization and arrhythmic risk following rate changes.

The goal of our study is to quantify LV/RV heterogeneities in APD and APD rate adaptation in the human ventricles using *in vivo* electrophysiological recordings, and to investigate their pro-arrhythmic implications using *in silico* simulations. We hypothesized that the human ventricles exhibit LV/RV heterogeneity in both APD and APD rate adaptation, which result in increased interventricular heterogeneity in APD shortly after heart rate acceleration. In our study, LV/RV differences in APD and APD rate adaptation were quantified from *in vivo* electrograms obtained at 10 LV and 10 RV endocardial locations of 15 patients using two decapolar catheters. Ethical limitations prevent the *in vivo* investigation of the pro-arrhythmic consequences of these LV/RV heterogeneities in the patients. We therefore conducted a simulation study to extend the implications of our *in vivo* findings. A human ventricular tissue model was constructed based on the *in vivo* recordings. Simulations were conducted using different stimulation protocols to systematically investigate the contribution of LV/RV heterogeneity in APD and APD rate adaptation to the pro-arrhythmic substrate following rate changes. Based on the higher incidence of in-hospital complications and post-discharge mortality in patients with ischemic regions near the LV/RV junction [23], [24], we hypothesize that LV/RV differences in APD rate adaptation contribute to increase the likelihood of unidirectional block at the LV/RV junction in the human ventricles following rate acceleration, which facilitates the establishment of reentry in the presence of an ischemic region.

## Methods

### Patients

Fifteen patients (4 females, 11 males; aged 35 to 72, median 61; see Table 1) with healthy ventricles were studied prior to radiofrequency ablation for supraventricular arrhythmias, as it is conventional to consider these patients as a group with normal ventricles [25]. The study, according to the principles expressed in the Declaration of Helsinki, was approved by the Guy's and St. Thomas' Hospital Ethics Committee, and written informed consent was obtained from all patients. Antiarrhythmic drugs were discontinued for 5 days before the study. Intrinsic rate for each patient was computed as the average of RR intervals over 1 minute before initiation of programmed pacing.

**Table 1:** Details of the patients in the study.

Patient	Diagnosis	Sex	Age	Intrinsic cycle length (ms)	Study cyclelength (ms)
**1**	AF	F	59	634±133	500
**2**	AF	M	52	844±136	500
**3**	AF	M	60	866±77	500
**4**	AF	F	72	679±266	500
**5**	AF	M	67	812±411	500
**6**	AF	F	69	945±155	500
**7**	AF	F	56	1091±311	500
**8**	AF	M	53	989±209	500
**9**	AF	M	68	567±28^*^	450**^†^**
**10**	AF	M	69	542±293	500
**11**	AF	M	35	855±451	500
**12**	AF	M	60	693±206	500
**13**	AF	M	61	723±32	600**^‡^**
**14**	AF	M	62	550±119^*^	500
**15**	AF	M	70	571±264	500
**Mean**	–	–	60.9	757.4	503.3
**STD**	–	–	9.5	173.0	29.7
**Median**	–	–	61.0	723.0	500.0

All patients were selected in the basis that they had healthy ventricles. AF = atrial fibrillation. Asterisks indicate patients recovering from a previous interrupted stimulation protocol. ^†^Patient 9 required a shorter cycle length of 450 ms in order to maintain capture, avoiding escape beats. ^‡^Patient 13 reported slight discomfort at the paced rate of 500 ms and was therefore paced at a longer cycle length of 600 ms.

### Data Acquisition

In-situ unipolar electrograms were recorded using two decapolar electrode catheters to quantify interventricular differences in APD and APD adaptation in the human ventricle. Both catheters were positioned in a base-to-apex orientation, one on the postero-inferior endocardial LV wall and the second on the antero-septal RV wall. Pacing was established from the RV apex at a pulse width of 2 ms and stimulus of strength 2×diastolic threshold. A period of 2 minutes was recorded from each patient following a sustained change in rate from their intrinsic rate (median 723.0 ms) to a faster cycle length (CL) of median 500 ms (see Table 1), in agreement with RR intervals observed clinically in exercise tests [22]. After the stabilization period, a standard restitution curve was constructed for each electrogram as previously reported [25].

### Signal Analysis

**Figure 1:**
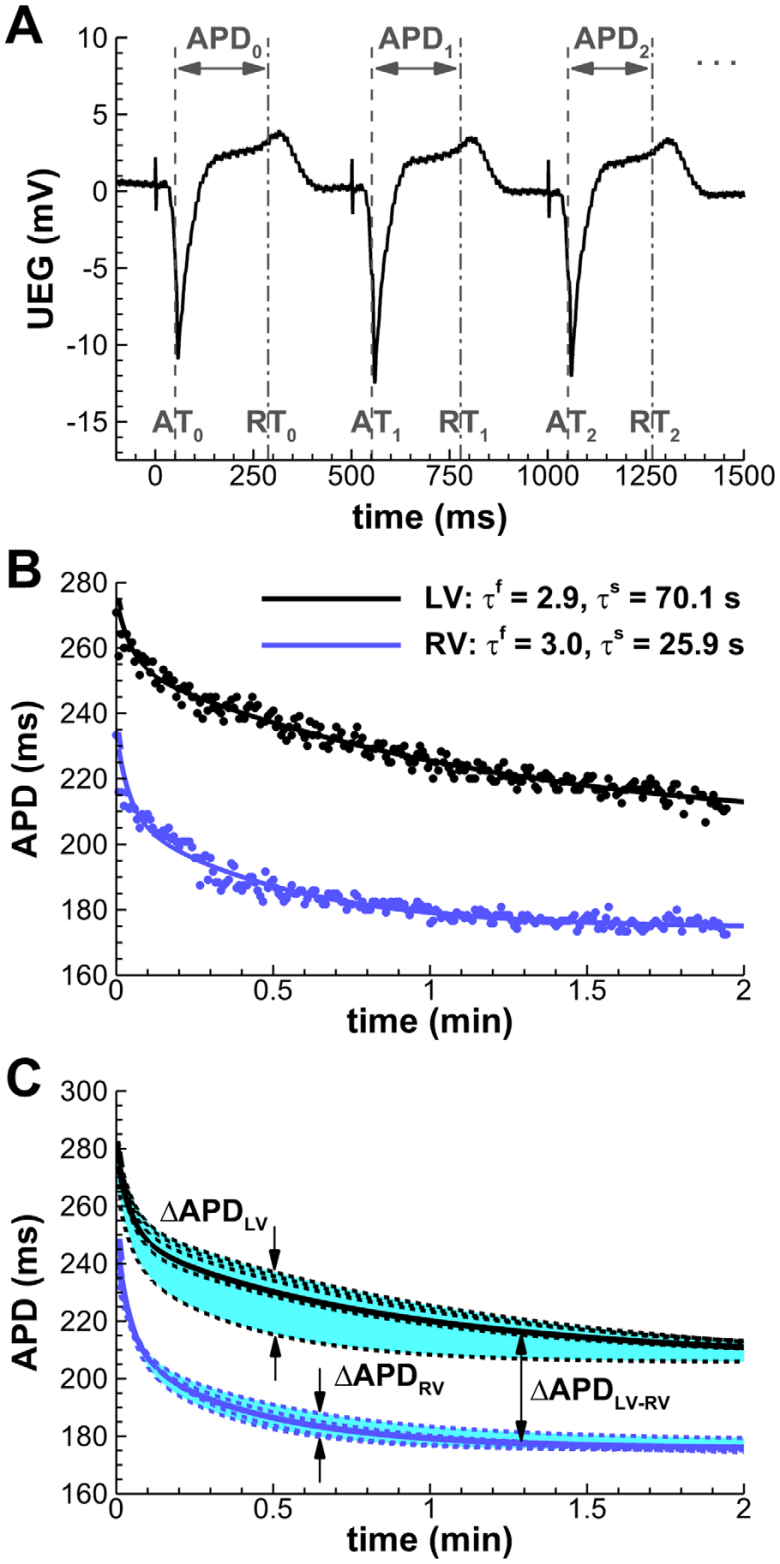
Quantification of intra- and interventricular APD dispersion (ΔAPD) from unipolar electrocardiograms. **A:** Automatic detection of activation and recovery times (AT/RT), and reconstruction of the APD series. The initiation of programmed pacing coincides here with *t = 0*. **B:** Estimation of fast (τ^f^) and slow (τ^s^) adaptation time constants by fitting the APD series to a double exponential decay (Patient 3, representative LV/RV mid-ventricular locations). **C:** Intraventricular ΔAPD is measured as the difference between the longest and shortest APD during adaptation (shaded areas). Solid lines indicate average LV/RV adaptations.

APDs were quantified as the activation-recovery intervals from each unipolar electrogram using the Wyatt method of analysis (Figure 1A), which has been validated following rigorous experimental and theoretical scrutiny [26]–[28]. The method was incorporated in an automated system, with manual verification [29]. Postprocessing of the APD series (Figure S1 and Text S1) was performed using custom-written routines in MATLAB (MathWorks, Natick, MA).

APD adaptation curves following rate acceleration were used to estimate the time constants of the fast (τ^f^) and slow (τ^s^) phases of APD adaptation for each electrogram by fitting each curve to a double exponential decay (Figure 1B). A robust least-squares algorithm (nlinfit, MATLAB Statistics Toolbox) was used to minimize the effects of outliers. The algorithm was validated against synthetic APD adaptation curves with different levels of noise-to-signal ratios, yielding satisfactory results in all circumstances (maximum relative τ^s^ error: 0.014±0.124; see Table S1).

Intraventricular APD dispersion in LV and RV (ΔAPD_LV_ and ΔAPD_RV_, respectively) was quantified for each patient as the difference between the longest and shortest APD in each ventricle at each time point (Figure 1C, shaded areas). Average differences between LV and RV were used to estimate interventricular APD dispersion (ΔAPD_LV-RV_), following the approach of Ramanathan *et al.* [14]. Mean LV and RV adaptation curves were computed for each patient as the average of all adaptation curves measured in each ventricle (Figure 1C, solid lines). ΔAPD_LV-RV_ was then quantified at each time point as the difference between these average APD adaptation curves.

### Statistical Analysis

Data are presented as mean±SD. The paired Student’s *t*-test was used to determine statistical significance in LV/RV properties.

### Human Ventricular Tissue Simulations

A computer simulation study was conducted to quantify the contribution of LV/RV differences in APD and APD adaptation characterized in the electrograms to the pro-arrhythmic substrate in the human ventricles following rate acceleration. Simulations of over 1000 beats were conducted using a two-dimensional human ventricular tissue model of 6×6 cm in size incorporating two regions describing RV and LV dynamics (similarly to the approach followed by Pandit *et al.* [10]). The long duration and number of simulations required in our study prevented the use of anatomically-based human ventricular models (real-time estimates based on 100 beats: 6.5 days per 3D simulation on 256 processors using Chaste [30], one of the most efficient and scalable world-wide finite-element solvers for computational cardiac electrophysiology; 3.5 hours per 2D simulation on 4 processors using our specific 2D spectral solver, see “Numerical Techniques”).

In all tissue simulations an isotropic monodomain model was used to simulate propagation of electrical excitation, with a diffusion coefficient (*D = 1.171* cm^2^/s) specifically calculated for human ventricular tissue [31]. The human virtual tissue was stimulated at the bottom edge perpendicular to the LV/RV junction with CL = 750 ms for 100 beats followed by acceleration to CL = 400 ms. Repolarization patterns were analyzed during rate acceleration occurring both suddenly and progressively during a 1 minute linear CL decay. In order to investigate vulnerability to reentry, ectopic stimulation was applied at the LV/RV junction at the time of maximum APD heterogeneity following rate acceleration. Simulations were conducted in the absence and presence of an inexcitable ischemic region at the LV/RV junction. The ischemic region was considered to be of 1 cm in radius and including non-excitable tissue, as previously described [32]–[34].

### Cellular Model of Human Ventricular Electrophysiology

Testing of our hypotheses requires a human ventricular action potential (AP) model in agreement with our *in vivo* recordings and which allows the independent alteration of APD and its rate dependent properties by varying specific parameters. This is made possible in our study by constructing a new version of the Bueno-Orovio–Cherry–Fenton human AP model [31], modified to capture APD adaptation dynamics as quantified in the electrograms. The model accounts for the sum of all transmembrane currents into three main categories (fast inward, slow inward, and slow outward currents):
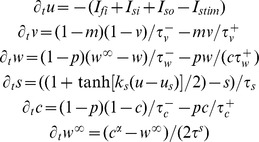
where



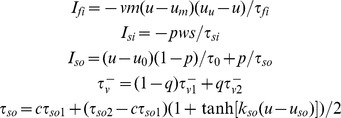
and



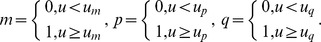



**Figure 2:**
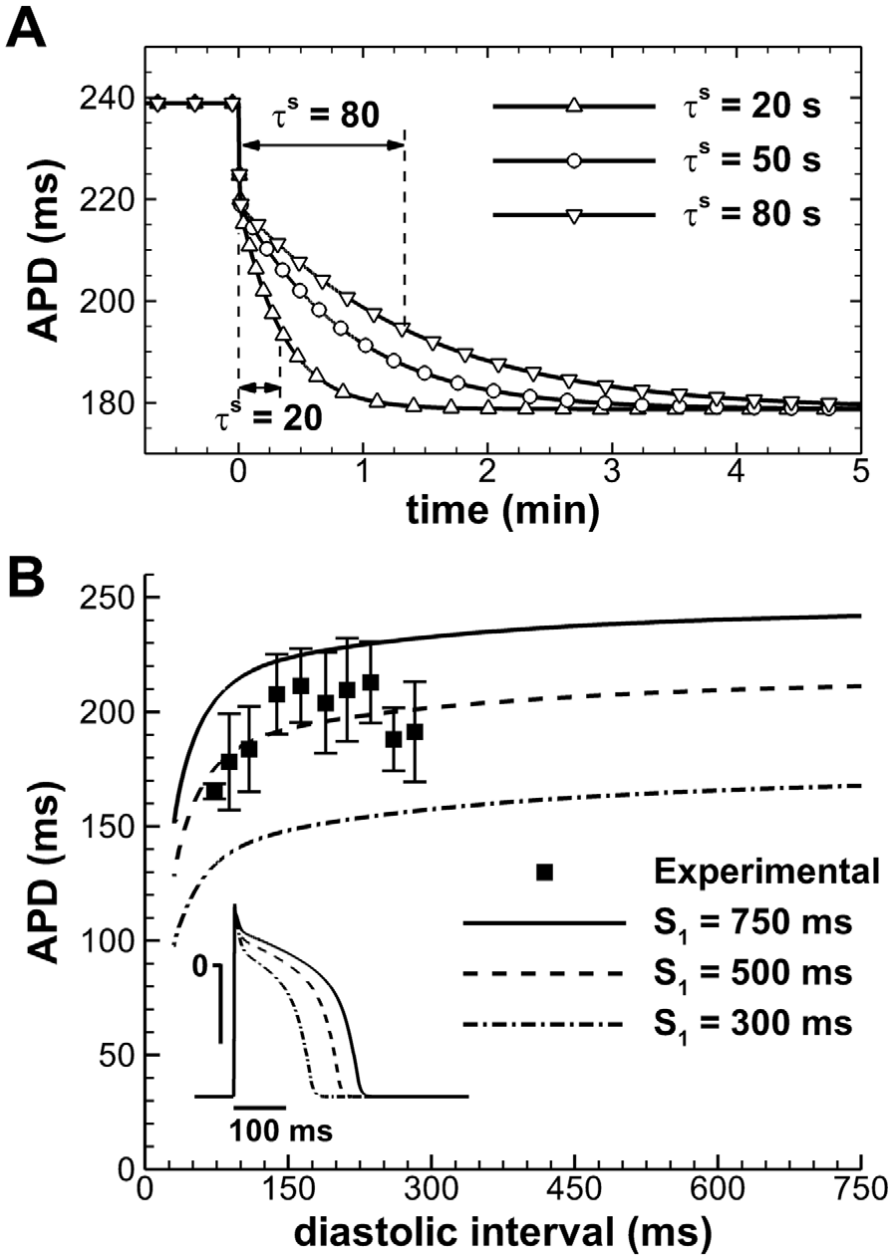
Electrophysiological properties of the human AP model. **A:** APD adaptation after a sustained change in rate from normal to fast pacing (750 to 400 ms), for different slow APD adaptation time constants. **B:** S_1_–S_2_ APD restitution at different S_1_ cycle lengths. Aggregated experimental restitution data at a CL = 500 ms is shown for comparison. Inset shows steady-state APs at the indicated CLs.

The voltage *u* is rescaled to physiological range by means of the mapping *V_m_ = 92u−83* (mV), with initial conditions given by *u = 0, v = 1, w = 1, s = 0, c = 1,* and *w^∞^ = 1.* The slow phase of APD adaptation is independently regulated by model parameter τ^s^ (Figure 2A). APD at sinus rhythm is controlled by the parameter τ_so1_, therefore allowing for different combinations of APD and APD adaptation to be represented by the human model.

Importantly, model parameters were adapted to reproduce human endocardial AP morphology, and APD and APD restitution properties measured in the *in vivo* unipolar electrograms, using a parameter-fitting algorithm [31]. This yielded the following choice of parameters: *u_0_* = 0, *u_u_* = 1.4, *u_m_* = 0.3, *u_p_* = 0.13, *u_q_* = 0.2, *τ_v1_^−^* = 23, *τ_v2_^−^* = 100, *τ_v_^+^* = 2, *τ_w_^-^* = 30, *τ_w_^+^* = 380, *τ_c_^-^* = 250, *τ_c_^+^* = 4000, *τ_so1_* = 40, *τ_so2_* = 1.2, *τ_fi_* = 0.115, *τ_o_* = 6, *τ_s_* = 2, *τ_si_* = 2.9013, *u_s_* = 0.9087, *k_s_* = 2.0994, *u_so_* = 0.65, *k_so_* = 2, *α* = 8.

Resting membrane potential of the model at normal rate (CL = 750 ms) is −83 mV, AP amplitude 130.7 mV, and maximum upstroke velocity 234.3 V/s, all in agreement with previously published physiological data [31]. APD was measured at a fixed threshold of −70 mV, representing 90% of repolarization. APD S_1_–S_2_ restitution curves were calculated at the center of one-dimensional cables of 2.5 cm length [31]. By varying the S_1_ CL used, a family of S_1_–S_2_ restitution curves was generated (Figure 2B). Comparison with aggregated experimental data obtained from the *in vivo* electrograms is also shown for further model validation. Conduction velocity was measured between neighboring points located at the center of the cable, yielding a maximal value of about 70 cm/s according to experimental results in human [35], and a dispersion of activation times similar to the ones observed experimentally (data not shown).

### Numerical Techniques

The simulation software was written in Fortran. The human AP model was integrated using a second-order Euler method in time, with constant time step of 0.025 ms, and a Fourier spectral method in space, allowing a space discretization of 0.03 cm due to the high-order convergence of these methods [36]. The accuracy of the numerical simulations was verified in one-dimensional cables by halving the time and space integration steps and verifying that this resulted in less than 5% change in conduction velocity [31].

## Results

### *In vivo* LV/RV Heterogeneity in APD and APD Adaptation Dynamics in Human Ventricles

**Figure 3:**
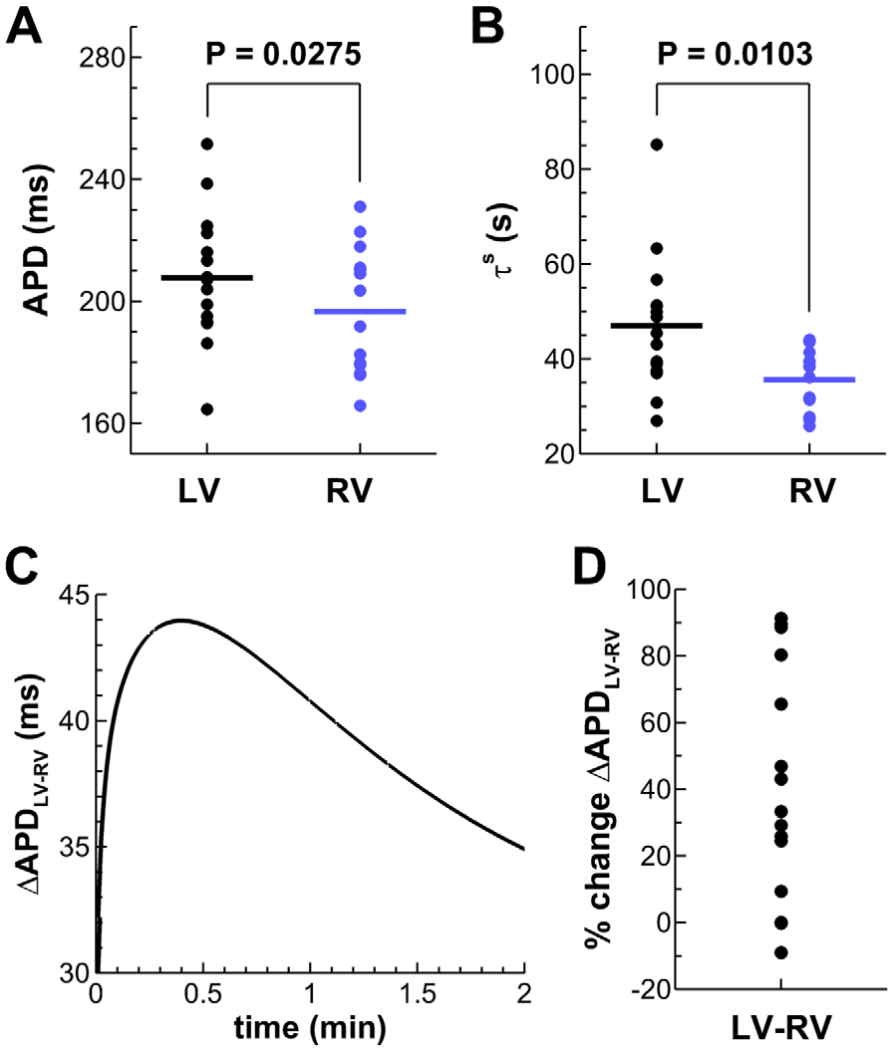
Functional LV/RV differences in the *in vivo* human heart. **A,B:** Unipolar electrograms revealed longer steady-state APDs at the study CL and slower APD adaptation dynamics in LV compared to RV. **C:** Transient increased LV/RV APD dispersion following rate acceleration in a representative patient (Patient 3). **D:** Percent of ΔAPD_LV-RV_ increase following rate acceleration for all patients of the study.

The analysis of the *in vivo* electrograms revealed significant LV/RV differences in steady-state APD and slow APD adaptation dynamics (*P<0.05*, paired Student’s *t*-test), whereas no statistical LV/RV differences were found for the time constant of the fast phase of APD adaptation (τ^f^ = 3.0±1.3 in LV, 2.6±0.8 s in RV). As shown in Figure 3, mean APD is longer in LV than in RV (panel A: 207.8±21.5 and 196.7±20.1 ms, respectively) and time constant of slow phase of APD adaptation is larger in LV than RV (panel B: τ^s^ = 47.0±14.3 and 35.6±6.5 s, respectively). The range of τ^s^ is also wider in LV than in RV, with τ^s^ = 26.9–85.2 in LV and τ^s^ = 25.8–44.0 s in RV. No significant correlations were observed between steady-state APD values and estimated time constants of adaptation (data not shown).

Following rate acceleration, LV/RV differences in APD (ΔAPD_LV-RV_) experienced an increase of up to 91% (mean±SD: 52.3±29.4%; median: 45.0%) of their initial value in 12 patients, following the pattern illustrated in Figure 3C. Time to peak maximum LV/RV APD dispersion was 26.7±20.1 s (median 24.5 s). Only 3 patients exhibited a monotonic decrease in ΔAPD_LV-RV_ following rate acceleration. The percent of ΔAPD_LV-RV_ increase over their initial value is shown for all patients in Figure 3D. Multiple linear regression analysis of the *in vivo* data showed a strong correlation (r^2^ = 0.90) of maximum ΔAPD_LV-RV_ with both initial ΔAPD_LV-RV_ and the LV/RV difference in slow adaptation time constant.

**Figure 4:**
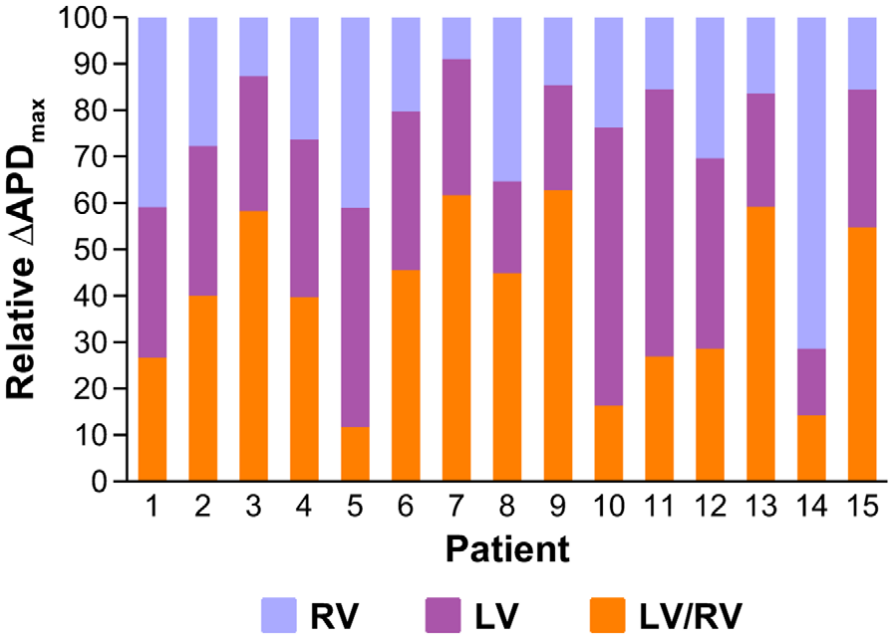
Comparison of maximum interventricular ΔAPD_LV-RV_ with intraventricular ΔAPD_LV_ and ΔAPD_RV_ for all patients in the study.

Figure 4 shows a comparison of values of maximum interventricular ΔAPD_LV-RV_ with intraventricular ΔAPD_LV_ and ΔAPD_RV_. Results show that ΔAPD_LV-RV_ is larger than both ΔAPD_LV_ and ΔAPD_RV_ in 9 patients, highlighting the importance of LV/RV differences in modulating ventricular heterogeneity. In 5 patients, ΔAPD_LV-RV_ is smaller than both ΔAPD_LV_ and ΔAPD_RV_, and in 1 patient ΔAPD_LV-RV_ is larger than ΔAPD_RV_ but smaller than ΔAPD_LV_.

### *In silico* Investigation of Pro-arrhythmic Consequences of LV/RV Heterogeneity in APD and APD Rate Adaptation

**Figure 5:**
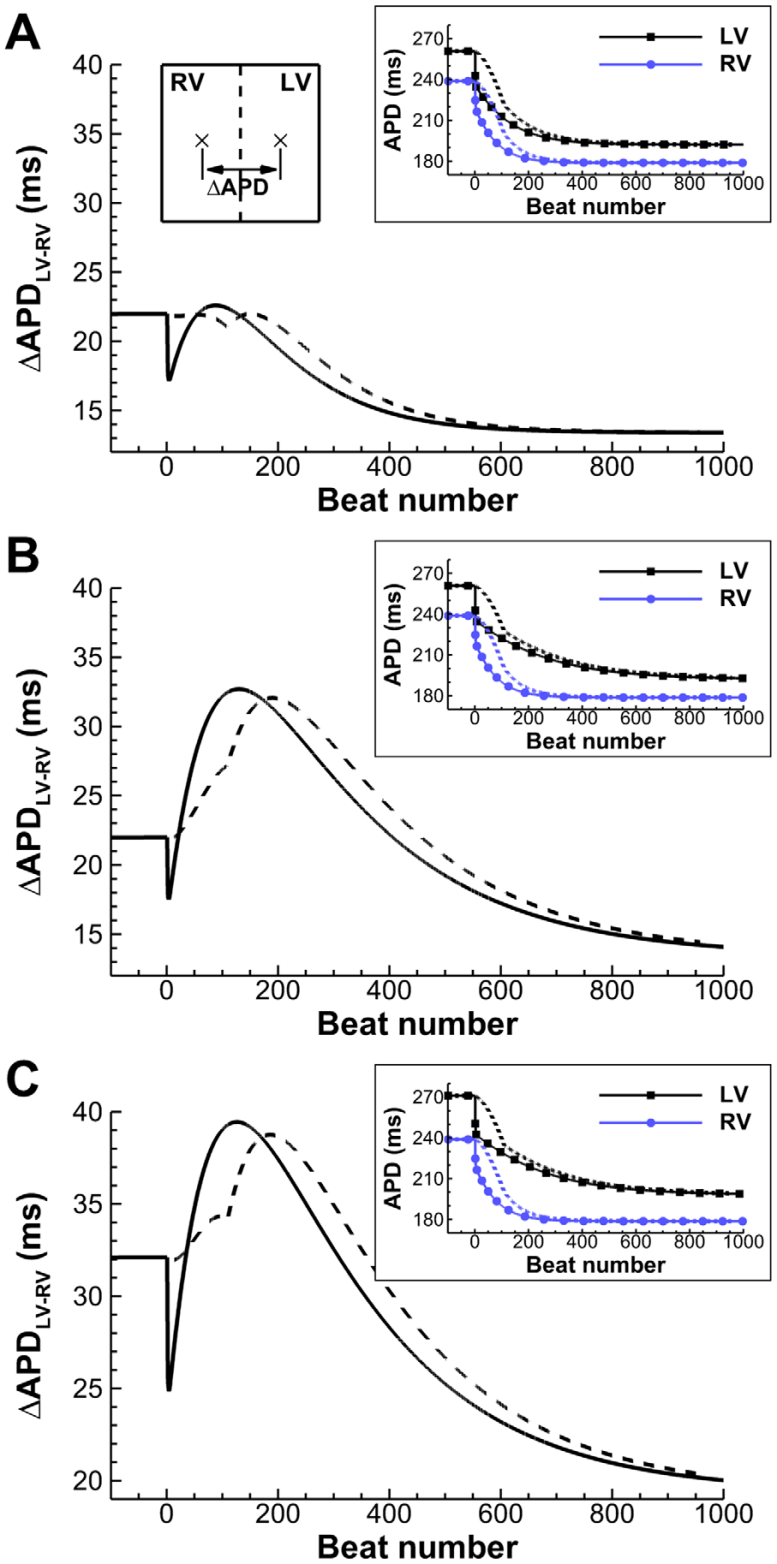
Time evolution of ΔAPD_LV-RV_ due to LV/RV differences in APD adaptation, after a sustained (solid) or a gradual (dashed) change in pacing rate (750 to 400 ms). A: Transient patterns of small ΔAPD_LV-RV_ develop under average conditions of slow APD adaptation. **B:** Conditions of protracted slow APD adaptation increase maximum amplitude and time window of the transient ΔAPD_LV-RV_ pattern. **C:** Conditions of larger ΔAPD_LV-RV_ at normal rate translate into a vertical shift of the transient pattern. Insets show LV/RV APD adaptation in each of the cases, for both stimulation protocols.

A simulation study was conducted to further investigate the implications of LV/RV heterogeneity in APD and APD adaptation shown in the *in vivo* recordings. Figure 5 shows the temporal evolution of ΔAPD_LV-RV_ between two points respectively located in the center of the RV and LV areas of the simulation domain, for different magnitudes of interventricular APD dispersion at normal heart rate and combinations of LV/RV adaptation time constants. Stimulation rate was increased from normal rate (CL = 750 ms) to fast pacing (CL = 400 ms) and either suddenly (solid lines) or linearly over a period of 60 s (dashed lines), the latter replicating rate changes occurring gradually. ΔAPD_LV-RV_ at normal and fast pacing in all scenarios was in range with the one reported in our *in vivo* electrograms, and also in agreement with the average ΔAPD_LV–RV_ of 32 ms reported at normal heart rate by Ramanathan *et al* [14].

In all the considered scenarios, sudden rate acceleration results in a sudden decrease in ΔAPD_LV-RV_ during the fast phase of APD adaptation, followed by a transient increase in ΔAPD_LV-RV_ before a gradual decrease towards the final ΔAPD_LV-RV_ value. For human tissue exhibiting moderate interventricular APD dispersion at normal heart rate (ΔAPD_LV-RV_ = 22 ms), and slow APD adaptation time constants of τ^s^ = 30 and 50 s in RV and LV (Figure 5A), maximum ΔAPD_LV-RV_ however remains very similar to its 22 ms value at normal rhythm. The introduction of larger LV/RV differences in APD adaptation dynamics (τ^s^ = 30 and 100 s in RV and LV; Figure 5B) results in a significant increase in maximum ΔAPD_LV-RV_ to 33 ms and also in a prolonged time window of increased ΔAPD_LV-RV_ of over 400 beats. An additional increase of interventricular APD dispersion at normal heart rate to ΔAPD_LV-RV_ = 32 ms (Figure 5C) results in similar qualitative patterns than in Figure 5B, but exhibiting a larger maximum ΔAPD_LV-RV_ reaching 40 ms. Furthermore, in the three cases, the transient initial decrease in ΔAPD_LV-RV_ following a sudden rate change, and attributed to the fast phase of adaptation, was not observed during progressive rate changes (dashed lines).

**Figure 6:**
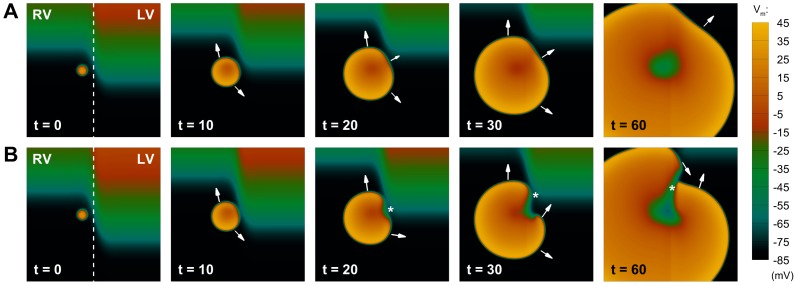
Development of unidirectional block due to transient patterns of interventricular APD dispersion. **A:** Under average conditions of slow APD adaptation (scenario A), the transient APD dispersion between both ventricles only affects wavefront propagation partially, and the ectopic stimulation excites the whole tissue as a regular beat. **B:** For conditions of protracted slow APD adaptation (scenario B), a larger interventricular APD dispersion is able to produce unidirectional block (*t = 20*, marked by an asterisk), leading to the initiation of reentry (*t = 30*), that subsequently develops in the tissue (*t = 60*). Colorbar denotes transmembrane potential (mV); times indicated since initiation of ectopic stimulation (ms).

Simulations were conducted to test the hypothesis that a transient increase in ΔAPD_LV-RV_ following rate acceleration increases the likelihood of unidirectional block and reentry in human ventricular tissue. An ectopic stimulus was applied near the LV/RV border at the time of maximum ΔAPD_LV-RV_, and subsequent dynamics were investigated. As shown in Figure 6A for scenario A, the maximum ΔAPD_LV-RV_ generated by moderate LV/RV heterogeneity in the slow phase of APD adaptation is not able to produce conduction block following the ectopic excitation (*t = 20*). The ectopic wavefront propagates as an almost circular pattern with its curvature only affected in a small region of the LV (*t = 30* to *60*).

In Figure 6B, however, the increased LV/RV heterogeneity in APD adaptation (scenario B) results in a larger transient dispersion of repolarization, which provides the substrate for the development of effective conduction block in the tissue (*t = 20*, asterisk), setting the stage for initiation of reentry (*t = 30*). However, the two wave fronts eventually merge, producing an excitation pattern similar to the one displayed in Figure 6A, with no wave-break or sustained reentry present in the tissue. Similar behavior was observed for conditions of increased ΔAPD_LV-RV_ at normal heart rate as considered in scenario C (data not shown). Therefore, our results suggest that heterogeneous APD adaptation dynamics between LV and RV, as reported in the *in vivo* electrograms, favor unidirectional block of propagation. However, additional conditions are required for the establishment of reentrant circuits. Our simulation results are therefore consistent with the lack of ventricular arrhythmias in the patients considered in this study, even considering larger LV/RV differences in rate adaptation than the ones reported experimentally (Figure 3B).

**Figure 7:**
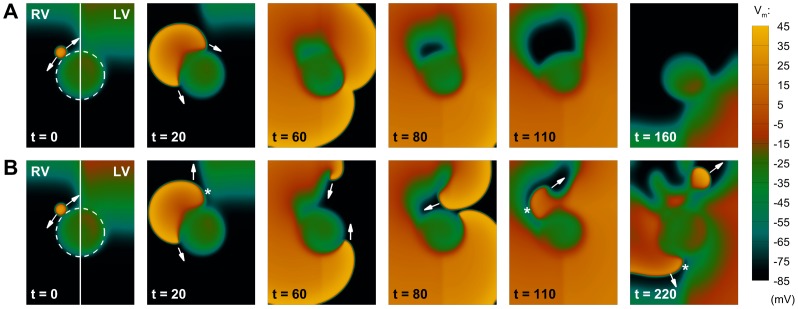
Interaction of transient patterns of interventricular APD dispersion with structural defects of the tissue. The dashed line indicates an inexcitable region in the LV/RV junction. **A:** Under average conditions of slow APD adaptation (scenario A), interventricular APD dispersion is not able to produce conduction block, and the extra-stimulus proceeds circumventing the inexcitable area. **B:** For conditions of protracted slow APD adaptation (scenario B), the top part of the extra activation now finds a region of unidirectional block due to a larger APD dispersion (*t = 20*, marked by an asterisk). The wavefront therefore moves upwards, eventually developing into a reentrant wave (*t = 60*). Since the bottom part of the excitation has been circumventing the obstacle, the top reentrant wave can now proceed in the tissue (*t = 80*), finding new areas of conduction block (*t = 110*), and finally producing wave-break (*t = 220*). Figure annotation as in Figure 6.

Given the high incidence of arrhythmic events in the presence of ischemic regions at the LV/RV junction [23], [24], we introduced the presence of an inexcitable region in our 2D model as shown in Figure 7 (dashed line). Ectopic stimulation was applied close to the ischemic border [37]. Figure 7A shows that, for modest LV/RV heterogeneity in APD adaptation (scenario A), a small dispersion of repolarization is again unable to produce conduction block (*t = 20*), and the ectopic stimulus circumvents the injured region with a normal excitation pattern (*t = 60* to *160*). However, as shown in Figure 7B, increasing LV/RV heterogeneity in rate adaptation (scenario B) results in effective unidirectional block in the LV following ectopic stimulation (*t = 20*), with the establishment of a reentrant circuit facilitated by the scar region (*t = 60*). Importantly, this irregular excitation pattern self-perpetuates in the tissue, eventually finding new areas of conduction block (*t = 110*), and ultimately producing wave-break and fibrillatory-like activity (*t = 220*).

An appropriate timing of the extra stimulus depending on ectopic location was required in our computer simulations in order to yield establishment of reentry. This is in line with previous studies highlighting the existence of a vulnerable window for reentry following ectopic stimulation [38], [39]. Hence, multiple combinations of ectopic timing and location could potentially produce similar dynamics as those reported in Figure 7 (data not shown). These findings are also in agreement with previous experimental results [40], highlighting the role of the time of arrival of the premature wavefront at the distal side of the line of block in determining the occurrence of reentry.

Our results therefore show that LV/RV heterogeneity in rate adaptation facilitates unidirectional block and initiation of reentry at the interventricular junction. Previous studies have suggested a possible role of the slow phase of APD adaptation in the transition from ventricular tachycardia to ventricular fibrillation, by modulating wave-break [41], [42]. We conducted further investigations to determine whether fast or protracted slow APD adaptation per se (rather than LV/RV differences) modulates the stability of reentrant rotors and wave-break. Over 200 tissue simulations were conducted under different scenarios, including homogeneous and linear gradients in APD adaptation and varying restitution slope steepness, as described in further detail in the Supplemental Material. In summary, our results show that the dynamics of the slow phase of APD adaptation do not modulate wave-break during reentry, but in contrast, this is primarily regulated by the steepness of APD restitution (Figure S2). Therefore, our simulation study identifies LV/RV heterogeneity in APD adaptation, rather than the dynamics of the slow phase of APD adaptation per se, as an important contributor to the substrate of reentrant arrhythmias in the human ventricles following rate acceleration.

## Discussion

In the clinical scenario, reentrant arrhythmias are generally considered to be multifactorial in origin, whereby a number of factors combine to generate an appropriate trigger and substrate at a given moment. In order to achieve as complete a mechanistic picture as possible, it is important to identify all the potential individual components.

Electrophysiological recordings from multiple LV and RV endocardial sites, acquired *in vivo* from humans with normal ventricles, exhibit significant LV/RV heterogeneity in APD and APD rate adaptation dynamics. In most patients, LV/RV APD heterogeneity transiently increases following rate acceleration due to LV/RV heterogeneity in rate adaptation. Computer simulations further demonstrate the importance of LV/RV heterogeneity in APD rate adaptation in increasing ventricular dispersion of repolarization following changes in rate and facilitating unidirectional block at the LV/RV junction. In structurally-normal ventricles, the transient increase in LV/RV APD heterogeneity following rate acceleration is insufficient for the establishment of a reentrant circuit. However, in the presence of an ischemic region at the LV/RV junction, LV/RV heterogeneity in APD and APD rate adaptation promotes initiation and establishment of reentrant circuits and their degeneration into fibrillatory activity, due to unidirectional block at the LV/RV junction.

Our *in vivo* study reports longer APDs in LV than in RV in the human ventricles (Figure 3A), in agreement with previous studies in canine [11], [12] and in non-invasive imaging of the human ventricles [14]. Our data also report for the first time slower APD adaptation dynamics in LV than in RV in the human ventricles (Figure 3B). Only a limited number of animal studies have previously suggested the existence of spatial heterogeneities in the slow phase of APD adaptation [43], [44]. Our data are thus in agreement with the reported slower APD adaptation in LV compared to RV on the epicardial surface of rabbit hearts [43].

Importantly, our *in vivo* data also show that LV/RV APD heterogeneity transiently increases following rate acceleration in most of the patients (Figures 3C–D). Maximum LV/RV heterogeneity in APD is correlated with both LV/RV heterogeneity in baseline APD and APD rate adaptation, supporting the importance of heterogeneity in APD rate adaptation dynamics in modulating dispersion of repolarization in the human ventricles. This finding is further supported by our simulation results, which show that the transient increase in LV/RV APD heterogeneity also occurs when rate acceleration occurs gradually rather than suddenly (Figure 5).

According to our simulation study, a transient increase in LV/RV APD heterogeneity following rate acceleration promotes unidirectional block, but it is insufficient for the establishment of reentrant circuits (Figure 6). This is consistent with the lack of arrhythmic events in the group of patients evaluated. However, our simulations suggest that the transient increase in LV/RV APD dispersion caused by heterogeneous APD adaptation can act in synergy with a pro-arrhythmic substrate (such as an ischemic region) to promote conduction block, reentrant activity and wave-break, potentially leading to the initiation of ventricular fibrillation (Figure 7B). Based on the high incidence of arrhythmic events and post-discharge mortality in patients with ischemic regions at the LV/RV junction [23], [24], the location of the infarcted area was prescribed in our computational study at the interventricular interface. Although different mechanisms than LV/RV differences might be involved in the establishment of reentrant arrhythmias for ischemic regions located outside this functional boundary, our findings provide supporting evidence of the important role of these interventricular differences in promoting the occurrence of arrhythmic events following rate acceleration [15]–[17], particularly in patients with protracted rate adaptation [21], [22].

Our investigations suggest the need to conduct further clinical and experimental investigations to confirm the importance of interventricular heterogeneity in APD rate adaptation for risk stratification in post-infarcted patients. Ideally, a long term follow-up study should be performed to determine possible correlations between LV/RV heterogeneities in slow adaptation, location and extension of the infarcted area, and the number of sudden cardiac deaths and arrhythmic episodes in these patients. However, the electrophysiological examination of ischemic or post-infarcted patients is usually challenging, due to the high risk of inducibility of ventricular fibrillation during examination in this group of patients. A possible way to circumvent these limitations is the use of clinical effort tests, and estimate APD rate adaptation heterogeneities from body surface ECG biomarkers associated to global dispersion of repolarization and its adaptation, such a QT or T-wave peak-to-end adaptation [21], [45].

The ionic mechanisms responsible for LV/RV heterogeneity in APD and APD rate adaptation were not investigated in our study due to the impossibility of conducting the recordings *in vivo*. Previous studies have shown that whereas the human ventricular APD is modulated by a number of repolarization currents [46], [47], the slow phase of APD adaptation is primarily determined by Na^+^ dynamics, and the Na^+^/K^+^ pump in particular [20]. It is therefore likely that the LV/RV differences reported in our study could be caused by heterogeneity in a number of ionic currents such as the I_to_ and I_Ks_ currents as reported in the canine ventricle [8], [11], and importantly heterogeneity in Na^+^/K^+^ pump activity as experimentally reported in the rat ventricles [48]. Further experiments in human using Western immunoblots could be used to quantify expressions of Na^+^/K^+^ pump and other proteins in the LV and RV to shed light into the ionic mechanisms underlying the LV/RV differences in APD and APD adaptation, characterized in our *in vivo* electrograms.

### Study Limitations

Electrograms were acquired under RV apical pacing from patients undergoing interventional procedures for atrial arrhythmias, the intrinsic rhythm in the majority of cases being atrial fibrillation. We can not therefore exclude an influence of these conditions in our results. Furthermore, due to associated practical challenges, our recording sites did only cover a reasonably-sized area within the ventricles. Hence, it would be reasonable to assume that over the whole endocardial surface there would be regions of greater and lesser APD heterogeneity.

Due to ethical constraints, electrograms were recorded following rate acceleration from intrinsic rhythm to CL = 500 ms, and thus the role of CL in determining adaptation dynamics was not investigated in the patients. Studies in isolated rabbit and guinea pig myocytes showed time constants of the slow phase of APD adaptation to decrease linearly with decreasing CL [49]. However, this linear dependence may not be present in tissue with intact cell-to-cell coupling, where adaptation time constants were found to be approximately constant for both large and small changes in CL [43].

In our computer simulations, a 2D model of human ventricular tissue with an idealized inexcitable region was used to test our hypothesis, taking into account the high computational cost associated with the simulations. Therefore, when rendered possible by improvements in computational power, further studies should evaluate the role of LV/RV heterogeneity in modulating the pro-arrhythmic substrate, where additional factors such as 3D structure of the human ventricles are taken into account.

### Conclusions

We report for the first time *in vivo* LV/RV heterogeneity in APD and, in particular, APD adaptation dynamics in the human ventricle, which are responsible for a transient increase in APD dispersion following rate acceleration. Our *in silico* investigations suggest that patients with LV/RV heterogeneous APD adaptation dynamics might be at higher risk of developing arrhythmias (particularly following an ischemic event), due to a higher likelihood of conduction block, reentry and wave-break which could degenerate to ventricular fibrillation. Our combined *in vivo* and *in silico* study provides new insights that offer a mechanistic explanation of the increased risk of cardiac arrhythmias and sudden cardiac death in patients exhibiting protracted QT adaptation, emphasizing the importance of this biomarker in arrhythmic risk stratification.

## Supporting Information

Figure S1 **Unipolar electrocardiogram (UEG) postprocessing flowchart.**
(TIF)

Figure S2 **The slow phase of APD adaptation does not facilitate reentrant wave-break.** A: Sustained wave-break when reentry is initiated in a steep APD restitution region, with homogeneous slow time constant of APD adaptation (τ^s^ = 50 s). B: Stable reentry pattern after reentry initiation in a flatter APD restitution region, with linear apico-basal gradient in the slow time constant of APD adaptation (τ^s^ = 20–80 s). Times indicated since initiation of reentry (ms); colorbar denotes transmembrane potential (mV).
(TIF)

Table S1 **Calibration of the methodology for estimation of slow time constants of APD adaptation.** **Top:** Parameter sets used to generate the synthetic APD series. **Bottom:** Estimated slow time constants of APD adaptation (mean±SD) for different noise instantiations (*n = 100*). Compare results with the last row of the top part of the table.
(DOC)

Text S1 **Expanded methods and results.**
(DOC)
