# Supplementary figures and images for: In Vivo Human Left-to-Right Ventricular Differences in Rate Adaptation Transiently Increase Pro-Arrhythmic Risk following Rate Acceleration
Source: PLoS One. 2012 Dec 20;7(12):e52234. doi: 10.1371/journal.pone.0052234 (PMC3527395; doi:10.1371/journal.pone.0052234)

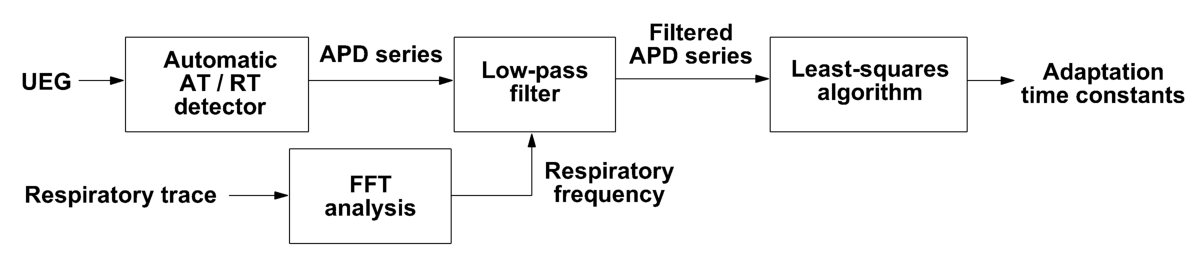

Supplement: Figure S1 — Unipolar electrocardiogram (UEG) postprocessing flowchart. (TIF) [file pone.0052234.s001.tif]

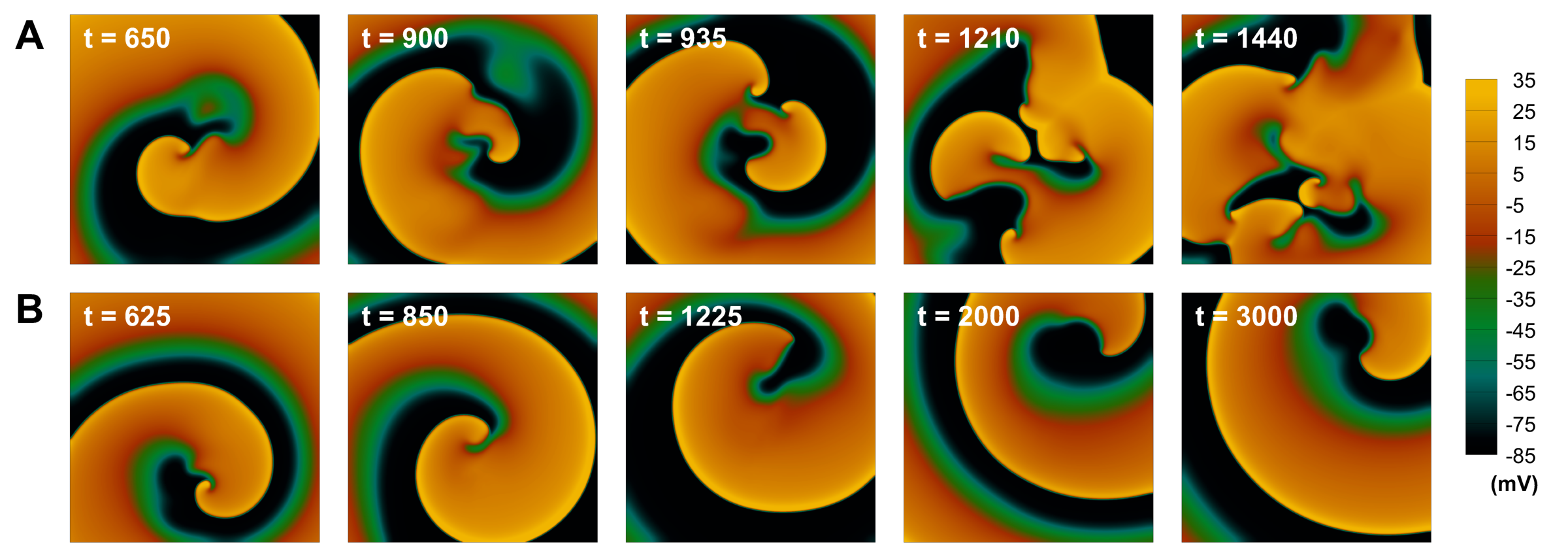

Supplement: Figure S2 — The slow phase of APD adaptation does not facilitate reentrant wave-break. A: Sustained wave-break when reentry is initiated in a steep APD restitution region, with homogeneous slow time constant of APD adaptation (τs = 50 s). B: Stable reentry pattern after reentry initiation in a flatter APD restitution region, with linear apico-basal gradient in the slow time constant of APD adaptation (τs = 20–80 s). Times indicated since initiation of reentry (ms); colorbar denotes transmembrane potential (mV). (TIF) [file pone.0052234.s002.tif]
