# Supplementary material for: In Vivo Human Left-to-Right Ventricular Differences in Rate Adaptation Transiently Increase Pro-Arrhythmic Risk following Rate Acceleration
Source: PLoS One. 2012 Dec 20;7(12):e52234. doi: 10.1371/journal.pone.0052234 (PMC3527395; doi:10.1371/journal.pone.0052234)
Supplement: Table S1 — Calibration of the methodology for estimation of slow time constants of APD adaptation. Top: Parameter sets used to generate the synthetic APD series. Bottom: Estimated slow time constants of APD adaptation (mean±SD) for different noise instantiations (n = 100). Compare results with the last row of the top part of the table. (DOC) [file pone.0052234.s003.doc]

| Site | 1 | 2 | 3 | 4 | 5 | 6 | 7 | 8 | 9 | 10 | | |
| --- | --- | --- | --- | --- | --- | --- | --- | --- | --- | --- | --- | --- |
| **Ai (ms)** | 180 | 185 | 190 | 185 | 190 | 195 | 200 | 195 | 200 | 190 | | |
| **Bi (ms)** | 20 | 25 | 30 | 25 | 30 | 35 | 20 | 40 | 30 | 30 | | |
| **Ci (ms)** | 20 | 15 | 10 | 25 | 25 | 20 | 15 | 30 | 10 | 30 | | |
| **Di (ms)** | 2 | 1.5 | 1 | 0.75 | 2.5 | 1.5 | 1.75 | 3 | 2 | 0.5 | | |
| **τi^fast^ (s)** | 0.7 | 1.1 | 1.8 | 1.7 | 1.4 | 1.8 | 1.3 | 1.3 | 1.5 | 2 | | |
| **τi^slow^ (s)** | 20.6 | 22.2 | 24.8 | 28.4 | 33.0 | 38.6 | 45.2 | 52.8 | 61.4 | 71.0 | | |
|  |  |  |  |  |  |  |  |  |  |  |  |  |
| **σ = 0** | 20.51 | 22.12 | 24.71 | 28.27 | 32.91 | 38.59 | 45.11 | 52.71 | 61.32 | 70.94 | | |
| **σ = 1** | 20.49 ±0.89 | 22.29 ±0.86 | 24.72 ±0.71 | 28.34 ±0.99 | 32.94 ±0.86 | 38.62 ±0.88 | 45.03 ±1.52 | 52.79 ±1.06 | 61.17 ±1.81 | 71.31 ±2.43 | | |
| **σ = 2** | 20.81 ±2.56 | 22.21 ±1.58 | 24.77 ±1.46 | 28.09 ±1.62 | 32.86 ±1.62 | 38.43 ±1.62 | 45.40 ±3.23 | 52.80 ±1.89 | 61.50 ±3.26 | 70.08 ±4.34 | | |
| **σ = 3** | 20.64 ±3.22 | 22.02 ±2.43 | 24.81 ±1.80 | 27.96 ±2.58 | 33.03 ±2.88 | 38.52 ±2.18 | 45.73 ±4.81 | 53.71 ±3.49 | 61.70 ±5.59 | 70.17 ±6.38 | | |
| **σ = 4** | 20.81 ±3.67 | 22.28 ±2.61 | 24.87 ±2.52 | 27.67 ±3.14 | 33.25 ±2.92 | 38.57 ±3.07 | 45.17 ±6.68 | 52.87 ±4.33 | 61.31 ±5.90 | 72.01 ±8.85 | | |
