# Supplementary material for: In Vivo Human Left-to-Right Ventricular Differences in Rate Adaptation Transiently Increase Pro-Arrhythmic Risk following Rate Acceleration
Source: PLoS One. 2012 Dec 20;7(12):e52234. doi: 10.1371/journal.pone.0052234 (PMC3527395; doi:10.1371/journal.pone.0052234)
Supplement: Text S1 — Expanded methods and results. (DOC) [file pone.0052234.s004.doc]

**expanded METHODS**

*Postprocessing flowchart of in vivo unipolar electrograms*

The postprocessing flowchart for the estimation of time constants of APD rate adaptation is illustrated in Supplemental Figure S1. For each beat of programmed pacing, APD was computed as the difference between recovery and activation times (RT/AT), extracted from the unipolar electrograms (UEG) using an automated system [1]. Missing beats in the APD series were recovered by linear interpolation at sample intervals determined by the period of the paced heart cycle length. Respiration was simultaneously measured during the adaptation response using a custom-adapted tension sensor fixed to a freely-expandable band placed around the patient's chest/abdomen [2], and its frequency estimated by means of FFT analysis. The APD series was then low-pass filtered at the estimated respiratory frequency to mitigate electrophysiological manifestations of breathing patterns [2]. Time constants of APD rate adaptation were then calculated by fitting each APD series to a double exponential decay, using a robust least-squares algorithm (nlinfit, MATLAB Statistics Toolbox) to minimize the effects of outliers in the distribution.

*Signal analysis validation*

In order to validate the above presented methodology for the estimation of the slow time constant of APD rate adaptation, our technique was calibrated against *“synthetic”* recordings in where 10 APD series were generated following the expression:

*APDi(t) = Ai + Bi·exp(-t/τi^slow^) + Ci·exp(-t/τi^fast^) + Di·sin(2πf_resp_ ·t) + Ni(0,σ)*

with *t* in [0,2] (min), *f_resp_* = 0.25 Hz. A normal distribution term of null mean and standard deviation *σ*, *Ni(0,σ)*, was also included in the series to further analyze the performance of the approach in the presence of different levels of noise. Different magnitudes of the fast, slow and oscillatory components were considered for each recording. The top part of Supplemental Table S1 resumes the parameter set used to generate the synthetic ARI series. In all circumstances, and even in the presence of high noise-to-signal ratios, the described methodology yielded satisfactory results in time constant estimation (less than 0.1 s of the true value without noise, 1 s in mean value for the highest noise-to-signal ratios considered).

**expanded results**

*Role of the slow phase of APD adaptation in modulating reentry wave-break*

In order to evaluate the role of the slow phase of APD adaptation in modulating wave-break, reentry was initiated in 2D tissue models by stimulating the bottom part of the domain with a stimulus of strength 2×diastolic threshold, and thus fractionating the planar wavefront when reaching the middle of the domain [3]. Tissue models were of 12x12 cm in size to allow reentry dynamics to fully develop without interfering with the domain boundaries. Same results were obtained in a subset of the simulations by applying a cross-field stimulation protocol to initiate reentry [4].

Reentry was initiated in steady-state conditions at two different cycle lengths (CL), representing steep (CL=750 ms) and flat (CL=400 ms) APD restitution, as illustrated in Figure 2 of the main manuscript. Rotor dynamics were studied for a time window of 5 s following initiation of reentry. To isolate the effects of the slow phase of APD adaptation (τ^s^), the tissue model was kept homogeneous in the rest of electrophysiological properties. The following scenarios in the slow phase of APD adaptation were considered: (i) homogeneous slow phase of APD adaptation throughout the entire tissue (τ^s^ varied from 20 to 100 s, τ^s^ steps of 10 s); (ii) linear apico-basal gradients in the slow phase of APD adaptation (apex τ^s^=20,40,60 to base τ^s^=60,80,100 s, and inverted).

In the simulation results, no effect could be attributed to the slow phase of APD adaptation but only to APD restitution in the break-up of the reentrant waves. Regardless of the slow adaptation dynamics (τ^s^ distribution), wave-break was always produced when reentry was initiated in the region of steep APD restitution (Supplemental Figure S2A), and rotors remained stable when initialized at flat APD restitution (Supplemental Figure S2B). Different APD and conduction velocity restitutions were also tested, yielding in all cases the same results. The whole simulation study comprised more than 200 tissue simulations.

**Supporting References**

1. Western DG, Taggart P, Hanson BM (2010) Real-time feedback of dynamic cardiac repolarization properties. Ann Intl Conf IEEE EMBS 114-117.
2. Western DG, Hanson BM, Gill J, Taggart P (2011) Oscillatory patterns of respiration: consequences for the stability and control of cardiac electrophysiology. Ann Intl Conf IEEE EMBS 1949-1952.
3. Mikhailov AS, Zykov VS (1991) Kinematical theory of spiral waves in excitable media: Comparison with numerical simulations. Physica D 52: 379-397.
4. Winfree AT (2000) Various ways to make phase singularities by electric shock. J Cardiovasc Electrophysiol 11: 286-289.
